# Comorbidities and Antithrombotic Treatment Pattern in Patients With Atrial Fibrillation

**DOI:** 10.3389/fneur.2022.761603

**Published:** 2022-03-04

**Authors:** Oh Young Bang, Siin Kim, Young Keun On, Myung-Yong Lee, Sung-Won Jang, Seongwook Han, Jaeyun Ryu, Seongsik Kang, Hae Sun Suh, Young-Hoon Kim

**Affiliations:** ^1^Department of Neurology, Samsung Medical Center, Sungkyunkwan University School of Medicine, Seoul, South Korea; ^2^College of Pharmacy, Kyung Hee University, Seoul, South Korea; ^3^Department of Cardiology, Samsung Medical Center, Sungkyunkwan University School of Medicine, Seoul, South Korea; ^4^Division of Cardiology, Department of Internal Medicine, Dankook University, Chung Nam, South Korea; ^5^Division of Cardiology, Department of Internal Medicine, Catholic University of Korea, Seoul, South Korea; ^6^Division of Cardiology, Department of Internal Medicine, Dongsan Hospital, Keimyung University School of Medicine, Daegu, South Korea; ^7^Pfizer Korea Ltd., Seoul, South Korea; ^8^Division of Cardiology, Department of Internal Medicine, Korea University, Seoul, South Korea

**Keywords:** stroke, systemic embolism, NOAC, warfarin, atrial fibrillation

## Abstract

**Objective:**

Non-vitamin K antagonist oral anticoagulants (NOACs) are proven alternatives to warfarin for preventing stroke in patients with non-valvular atrial fibrillation. We aimed to examine the treatment patterns and patient factors associated with the use of antiplatelet agents, warfarin, and NOACs in clinical practice.

**Methods:**

We conducted a retrospective cohort study using the Korean Health Insurance Review & Assessment Service database. Patients receiving antithrombotics were identified before and after the introduction of NOACs (from August 1, 2013 to December 30, 2014 and July 1, 2015 to November 30, 2016, respectively). Patients were included if they were aged ≥18 years, had an atrial fibrillation diagnosis, and had a CHA_2_DS_2_-VASc score ≥2. Treatment pattern was assessed by classifying patients into NOAC, warfarin, or antiplatelet users based on the first date of antithrombotic prescription. Clinical factors associated with the type of antithrombotics chosen were examined using logistic regression analyses.

**Results:**

We identified 129,465 and 196,243 patients before and after the introduction of NOACs, respectively. The proportion of antiplatelet users was 60.7 and 53.0% before and after the introduction of NOACs, respectively. The proportion of warfarin users was higher in patients with low HAS-BLED score, high CHA_2_DS_2_-VASc score, or stroke before the NOAC era. A similar trend was observed for NOAC and warfarin users after the introduction of NOAC. Compared with antiplatelets, warfarin and NOAC uses were significantly associated with CHA_2_DS_2_-VASc score and stroke, whereas presence of myocardial infarction (MI) and peripheral arterial disease were significantly associated with antiplatelets prescription. For comparisons between NOAC and warfarin, HAS-BLED and CHA_2_DS_2_-VASc scores showed significant associations with NOAC use, whereas comorbidities including MI were significantly associated with warfarin use.

**Conclusions:**

The treatment pattern of antithrombotics did not change with the introduction of NOACs. However, comorbidities served as an important factor in choosing treatment regardless of NOAC entry.

## Introduction

Large randomized controlled trials of patients with non-valvular atrial fibrillation (NVAF) have established that non-vitamin K antagonist oral anticoagulants (NOACs) are as effective as warfarin for preventing stroke/systemic embolism (S/SE) and are safer than warfarin regarding major bleeding (MB) and intracranial hemorrhage (1, 2), making NOACs the recommended first-line drug for stroke prophylaxis in patients with NVAF; hence, their use has grown dramatically worldwide (3–5).

Many patients with NVAF have one or more comorbidities. Approximately 20–40% of patients with atrial fibrillation (AF) present with coronary heart disease (CHD), whereas ~5–10% of patients undergoing percutaneous coronary intervention (PCI) have AF (6). In a pivotal trial of NOAC, one in four patients with AF was found to have had a prior PCI (7). Antithrombotic treatment patterns may differ depending on the presence of comorbidities. Moreover, the presence of comorbidities, such as stroke, CHD, and peripheral arterial disease (PAD), may affect treatment patterns of antithrombotics in patients with NVAF (8). Additionally, many patients with NVAF are prescribed multiple medications, and antiplatelet agents are widely used in clinical practice (9). In patients receiving oral anticoagulant (OAC) treatment for prevention of stroke, concomitant treatment with antiplatelets was shown to be associated with an increased rate of MB (10), which may affect treatment patterns of OACs in patients with NVAF.

For more appropriate use of OACs to prevent S/SE in NVAF patients, factors affecting treatment patterns of antithrombotics need to be evaluated. We hypothesized that comorbidity affects treatment patterns of antithrombotics in patients with NVAF, even after the introduction of NOACs. Therefore, we compared antithrombotic treatment patterns before and after the introduction of NOACs and examined the factors that affect treatment patterns, including comorbidities, such as stroke, myocardial infarction (MI), and PAD.

## Materials and Methods

### Study Design and Data Source

We conducted a retrospective cohort study using the Korean Health Insurance Review & Assessment Service (HIRA) database from January 1, 2007 to November 30, 2016. We explored the treatment patterns of antithrombotics and the clinical factors associated with the type of antithrombotics chosen for NVAF patients both before and after the introduction of NOACs in South Korea. We identified two separate groups of NVAF patients who received antithrombotics: during the first (from August 1, 2013 to December 30, 2014; before introduction of NOACs) and second intake periods (from July 1, 2015 to November 30, 2016; after introduction of NOAC). Patients were included in both groups if they had received antithrombotics during both periods. Antithrombotics included antiplatelets (aspirin, clopidogrel, ticagrelor, prasugrel, and ticlopidine), NOACs (apixaban, dabigatran, and rivaroxaban), and warfarin. Index date was the first date of antithrombotics prescription during the intake period.

The HIRA database includes patient-level information on diagnosis, treatment, procedure, and medication of ~50 million beneficiaries, which corresponds to 98% of the total population of South Korea. This is owing to the universal coverage of the National Health Insurance program (11). This study was exempt from ethical review from the Institutional Review Board of Pusan National University (PNU IRB/2019_101_HR).

### Study Population

Patients satisfying all of the following criteria were included in the study: (1) received antithrombotics during the intake period; (2) aged 18 years or older on the index date; (3) had more than one medical claim for AF within 6.5 years of the index date; and (4) had a CHA_2_DS_2_-VASc score ≥2 in the year before the index date. Patients were excluded from the study if they had medical claims for: (1) valvular AF or prosthetic heart valves within 1 year of the index date; (2) venous thromboembolism within 1 year of the index date; (3) hip/knee replacement surgery within 6 weeks of the index date; (4) end-stage chronic kidney disease, kidney transplant, dialysis, or pericarditis within 1 year of the index date; and (5) transient AF or cardiac surgery within 1 year of the index date. Patients who had been prescribed multiple OACs on the index date were also excluded from the study. Diagnosis, procedure, and medication codes used to define the study population are listed in Supplementary Table 1.

### Outcome Measures

For each of the two groups identified before and after the introduction of NOACs, we assessed the treatment patterns of antithrombotics and clinical factors associated with the choice of antithrombotic. To assess treatment patterns, patients prescribed NOACs or warfarin on the index date were classified as NOAC or warfarin users, respectively, regardless of antiplatelet co-prescription. Patients prescribed antiplatelets without NOACs or warfarin on the index date were classified as antiplatelet users. We examined the proportion of patients prescribed each type of antithrombotic based on CHA_2_DS_2_-VASc score, HAS-BLED score (Supplementary Table 2), and comorbidity. Comorbidities included stroke, MI, and PAD, which may affect the choice and pattern of anticoagulation therapy. Patients were regarded as having stroke if they had more than one diagnosis of ischemic stroke or transient ischemic attack (International Classification of Diseases, Tenth Revision code of I63, I69.3, or G45.9 as main or subdiagnosis codes) within 6.5 years of the index date. MI and PAD were defined by the diagnosis codes of the Charlson Comorbidity Index (CCI; Supplementary Table 3).

We examined the clinical factors associated with the choice of antithrombotics, such as age, sex, CHA_2_DS_2_-VASc score, HAS-BLED score, CCI, comorbidities (e.g., stroke, MI, and PAD), and medication use, such as non-steroidal anti-inflammatory drugs (NSAIDs), proton pump inhibitors (PPIs), H2-receptor antagonists, antiarrhythmics, digoxin, and statins.

### Statistical Analysis

Treatment pattern was analyzed descriptively and are presented as numbers and proportions. For sensitivity analysis, the change in antithrombotic treatment pattern was evaluated excluding overlapping patients in first and second intake periods. To examine the clinical factors associated with the choice of antithrombotics, we used a logistic regression to estimate odds ratios (ORs) and 95% confidence intervals (CIs). The SAS Enterprise Guide (version 6.1 M1; SAS Institute Inc., Cary, NC, USA) was used for statistical analyses. *P* < 0.05 was considered statistically significant.

## Results

### Baseline Characteristics of Study Population

**Figure 1:**
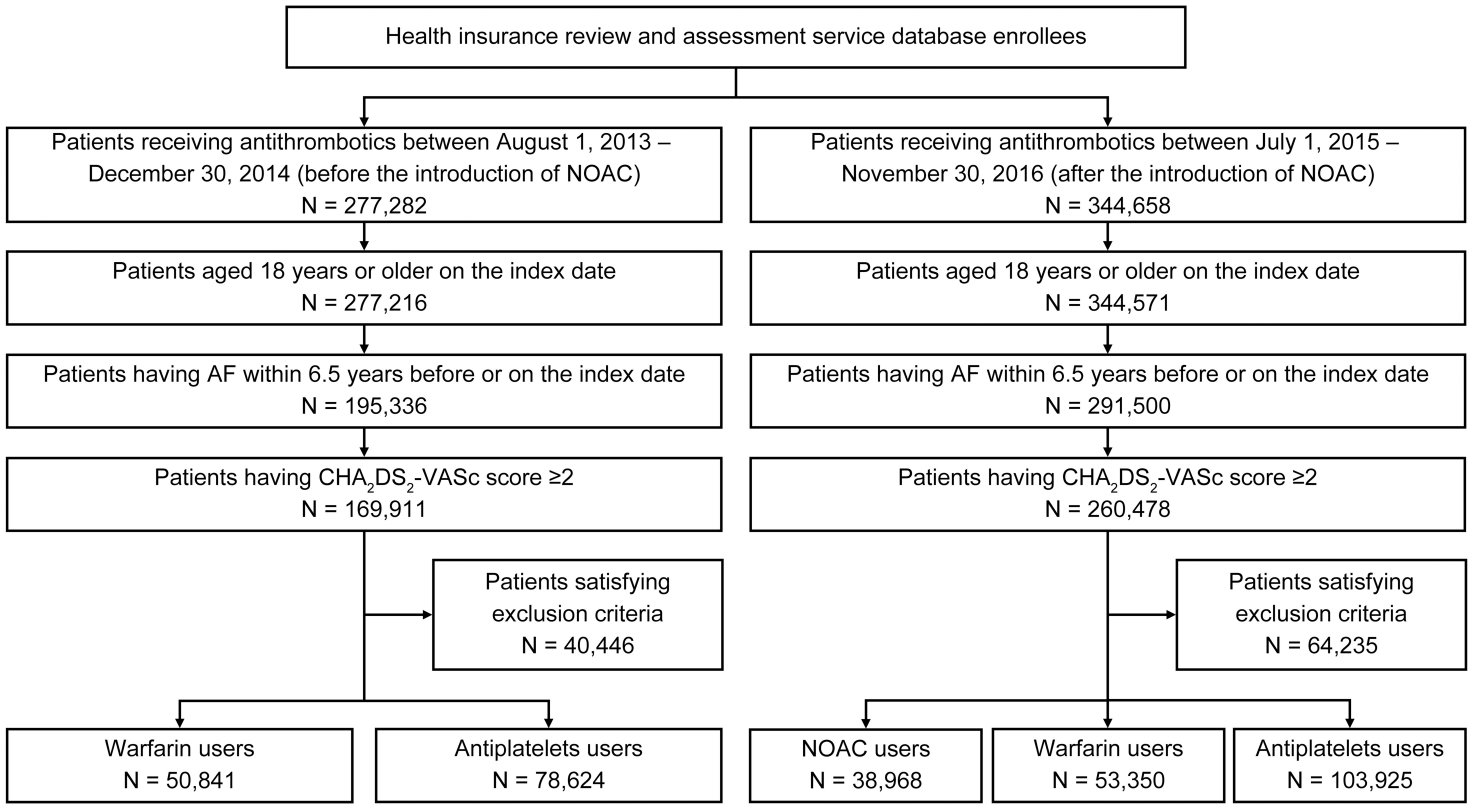
Patient selection flow diagram. AF, atrial fibrillation; NOAC, non-vitamin K antagonist oral anticoagulants.

We identified 129,465 and 196,243 patients for the first and second intake periods, respectively (Figure 1). Baseline characteristics were largely similar between the two groups (Table 1). For the first and second intake periods, mean ages were 70.6 and 71.9 years, respectively, and 43.8 and 44.0% were women, respectively. Mean CHA_2_DS_2_-VASc scores were 4.0–4.2. More than 80% of patients had a HAS-BLED score ≥3, which indicated that patients were at an increased risk of bleeding. For comorbidities, 39.1–40.3, 5.2–5.8, and 21.1–22.9% of patients had stroke, MI, and PAD, respectively.

**Table 1:** Baseline characteristics of study population.

**Characteristics**	**Patients identified before the introduction of NOAC**	**Patients identified after the introduction of NOAC**
	**(*n* = 129,465)**	**(*n* = 196,243)**
Age (years, mean, SD)	70.6 (9.7)	71.9 (10.3)
Female	56,718 (43.8%)	86,371 (44.0%)
**Insurance type**		
National health insurance	118,981 (91.9%)	180,698 (92.1%)
Medical aid	9,144 (7.1%)	14,593 (7.4%)
Veterans affairs	1,340 (1.0%)	952 (0.5%)
**CHA_2_DS_2_-VASc score**		
Mean, SD	4.0 (1.6)	4.2 (1.6)
2	24,999 (19.3%)	32,449 (16.5%)
3	29,497 (22.8%)	40,906 (20.8%)
4	29,305 (22.6%)	43,134 (22.0%)
≥5	45,664 (35.3%)	79,754 (40.6%)
**HAS-BLED**		
Mean, SD	3.4 (1.0)	3.4 (1.1)
0–2	23,585 (18.2%)	34,034 (17.3%)
≥3	105,880 (81.8%)	162,209 (82.7%)
CCI (mean, SD)	3.1 (2.1)	3.4 (2.3)
**Comorbidities**		
Stroke	50,633 (39.1%)	79,088 (40.3%)
Myocardial infarction	6,711 (5.2%)	11,480 (5.8%)
Peripheral artery disease	27,281 (21.1%)	44,918 (22.9%)
Bleeding	24,452 (18.9%)	40,612 (20.7%)
Hypertension	116,674 (90.1%)	174,980 (89.2%)
Diabetes mellitus	55,516 (42.9%)	88,563 (45.1%)
Congestive heart failure	54,141 (41.8%)	90,064 (45.9%)
COPD	50,899 (39.3%)	82,406 (42.0%)
Renal disease	4,957 (3.8%)	9,324 (4.8%)
**Medication use**		
NSAID	102,342 (79.0%)	153,950 (78.4%)
Antiplatelets	99,695 (77.0%)	139,763 (71.2%)
PPI	37,283 (28.8%)	70,458 (35.9%)
H2-receptor antagonists	82,644 (63.8%)	124,541 (63.5%)
Digoxin	36,741 (28.4%)	48,643 (24.8%)
Statin	61,707 (47.7%)	103,673 (52.8%)
Antiarrhythmics	51,711 (39.9%)	77,339 (39.4%)

*CCI, Charlson Comorbidity Index; COPD, chronic obstructive pulmonary disease; NOAC, non-vitamin K antagonist oral anticoagulants; NSAIDs, nonsteroidal anti-inflammatory drugs; PPIs, proton pump inhibitors; SD, standard deviation*.

### Treatment Pattern of Antithrombotics

**Figure 2:**
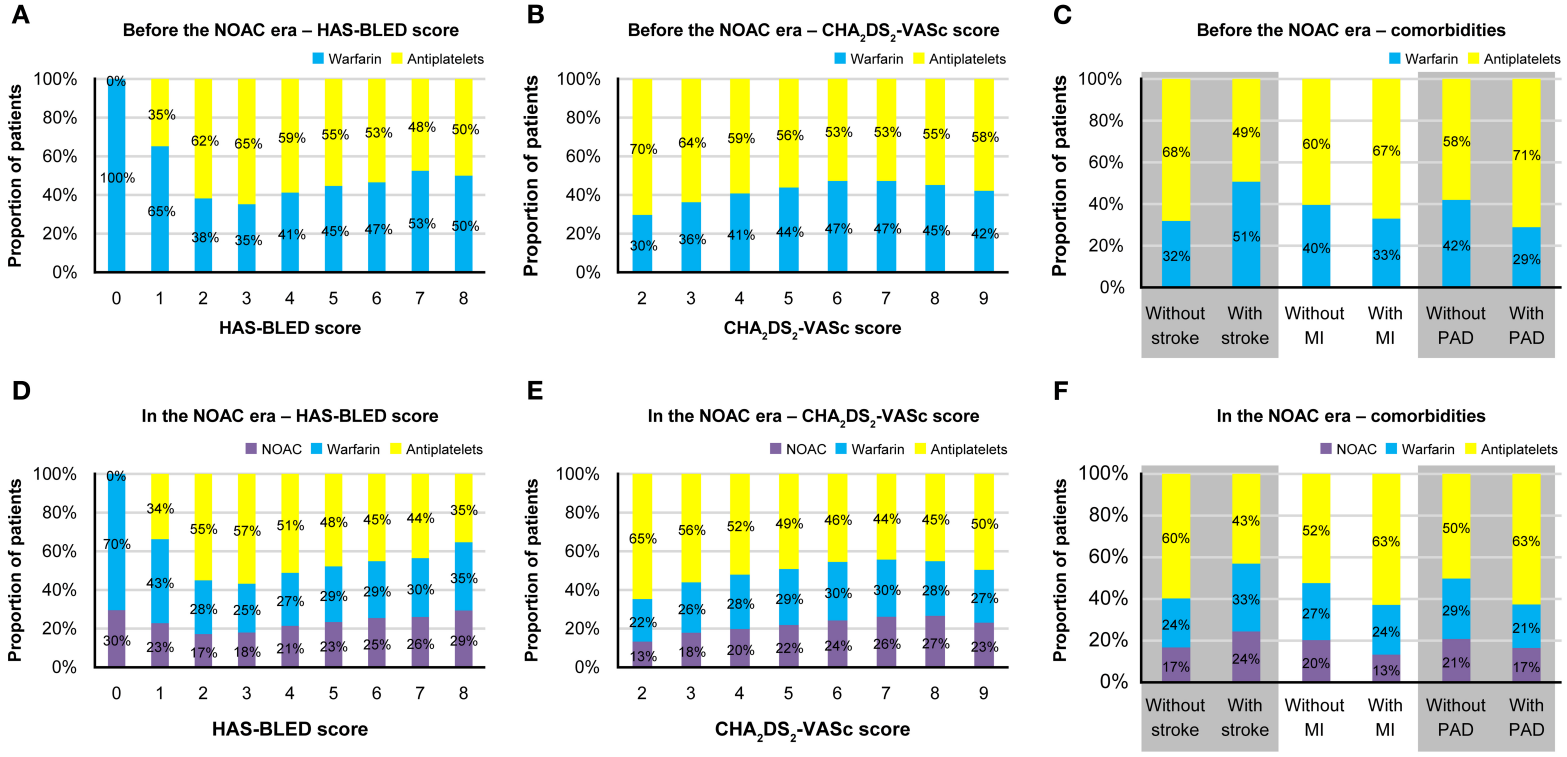
Treatment patterns of antithrombotics before and after the introduction of NOACs. Proportions of patients prescribed each type of antithrombotics before the NOAC introduction **(A)** according to the HAS-BLED score; **(B)** according to the CHA_2_DS_2_-VASc score; **(C)** according to the comorbidities, and after the NOAC introduction **(D)** according to the HAS-BLED score; **(E)** according to the CHA_2_DS_2_-VASc score; **(F)** according to the comorbidities. MI, myocardial infarction; NOAC, non-vitamin K antagonist oral anticoagulants; PAD, peripheral artery disease.

Before the introduction of NOACs, warfarin was preferred in patients with a HAS-BLED score of 0–1, and the proportion of warfarin users tended to increase with higher CHA_2_DS_2_-VASc scores (Figures 2A,B). Patients with stroke had a higher proportion of warfarin users than those without stroke, whereas the proportion of warfarin users was lower among patients with MI or PAD than those without MI or PAD (Figure 2C). Among warfarin users, 24.6% were co-prescribed antiplatelets. Among antiplatelet users, 11.8% were treated with more than one antiplatelet medication (i.e., dual or triple antiplatelet therapy).

The findings were relatively similar after the introduction of NOACs (Figures 2D–F). OACs (either warfarin or NOAC) were preferred among patients with HAS-BLED scores of 0–1, and the proportion of OAC users who used NOACs instead of warfarin numerically increased with increasing HAS-BLED score, from 30 to 46.4%. The proportion of OAC users tended to increase with CHA_2_DS_2_-VASc score, and the proportion of patients treated with NOACs among OAC users numerically increased with increasing CHA_2_DS_2_-VASc score, from 37.1 to 49.1%. The proportion of warfarin and NOAC users tended to be higher in patients with stroke and those without MI or PAD. Among NOAC and warfarin users, 20.3 and 21.1% were, respectively, co-prescribed antiplatelets, and 13.2% of antiplatelet users were treated with more than one antiplatelet.

When removing the patients included in both intake periods, treatment pattern was generally similar with the results of base case (Supplementary Figure 1).

### Clinical Factors Associated With the Choice of Antithrombotics

**Figure 3:**
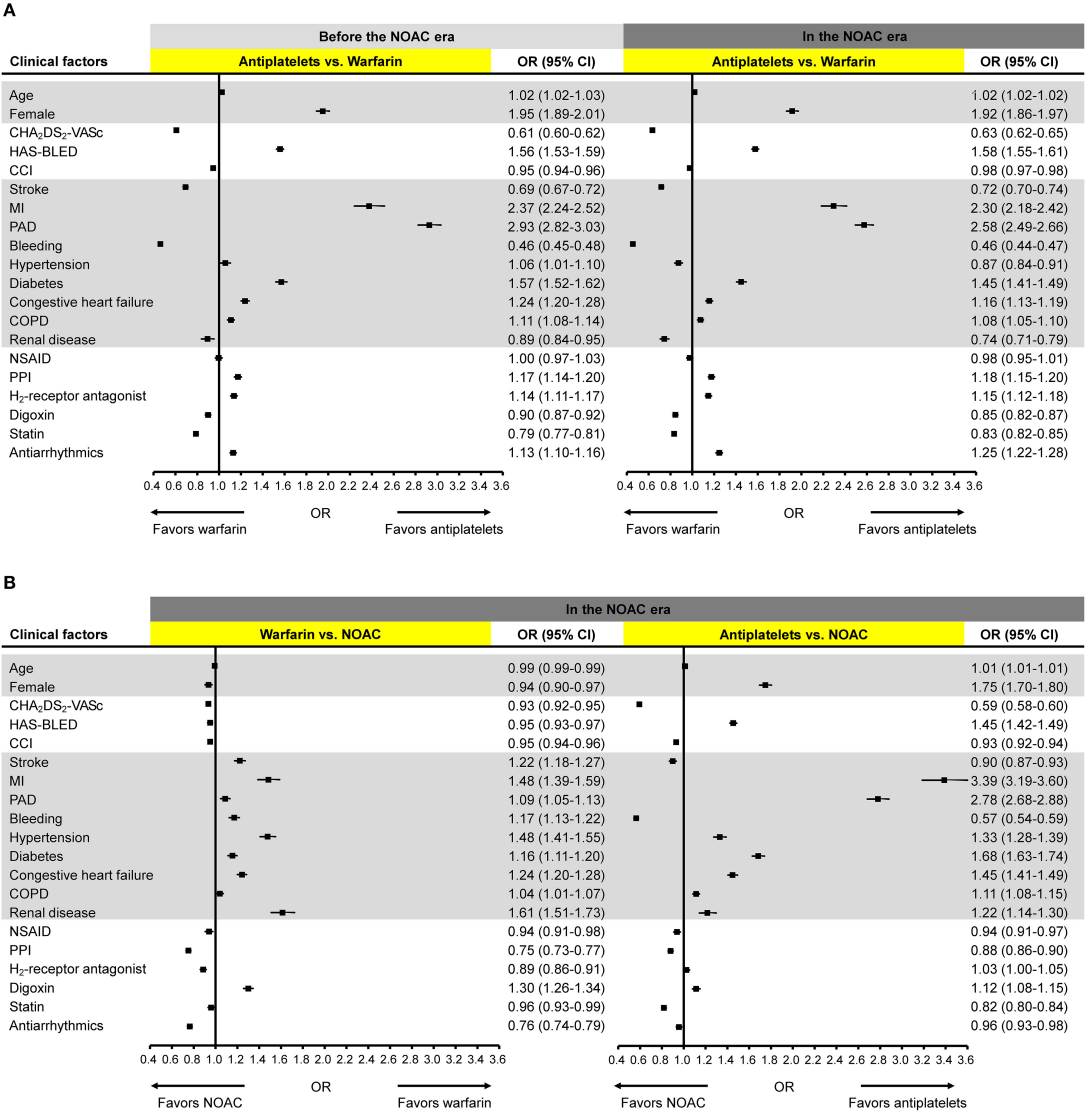
Clinical factors associated with the choice of antithrombotics. Odds ratios for the association of clinical factors with the choice of **(A)** antiplatelets vs. warfarin before and after the NOAC introduction; **(B)** warfarin vs. NOAC and antiplatelets vs. NOAC after the NOAC introduction. CCI, Charlson Comorbidity Index; CI, confidence interval; COPD, chronic obstructive pulmonary disease; MI, myocardial infarction; NOAC, non-vitamin K antagonist oral anticoagulants; NSAID, non-steroidal anti-inflammatory drug; OR, odds ratio; PAD, peripheral artery disease; PPI, proton pump inhibitor.

The change in ORs of clinical factors associated with the choice between warfarin and antiplatelets was small with the introduction of NOACs (Figure 3A). Age, female sex, HAS-BLED score, MI, PAD, diabetes, congestive heart failure, chronic obstructive pulmonary disease (COPD), PPI, H2-receptor antagonist, and antiarrhythmic were significantly associated with the use of antiplatelets, whereas CHA_2_DS_2_-VASc score, CCI score, stroke, bleeding, renal disease, digoxin, and statin were significantly associated with warfarin use, both before and after the introduction of NOACs.

After the introduction of NOACs, all clinical factors showed significant ORs with relatively small effect sizes when comparing prescription preference of warfarin and NOAC (Figure 3B). All comorbidities and digoxin use were significantly associated with warfarin use while all other clinical factors including HAS-BLED and CHA_2_DS_2_-VASc scores favored NOAC use. When compared with antiplatelets, NOACs were more likely to be used in patients with higher CHA_2_DS_2_-VASc score, higher CCI score, stroke, bleeding, NSAID, PPI, statin, or antiarrhythmics. Female sex, HAS-BLED score, MI, PAD, hypertension, diabetes, congestive heart failure, COPD, renal disease, and digoxin showed significant associations with the use of antiplatelets.

When comparing combined OAC and antiplatelet therapy with OAC monotherapy, MI showed the strongest association with combined therapy followed by statin, PAD, and HAS-BLED score (Supplementary Figure 2).

## Discussion

In our real-world population data, we found that patient treatment patterns of antithrombotics did not change significantly after the introduction of NOACs. OACs were not commonly used as recommended by the guidelines, and the proportions of antiplatelet users were 60.7 and 53.0% before and after the introduction of NOACs, respectively. Moreover, the factors affecting treatment patterns of antithrombotics remained the same. Stroke and CHA_2_DS_2_-VASc score were associated with the use of OACs, whereas female sex, MI and PAD were associated with the use of antiplatelets. When comparing NOACs with warfarin, higher HAS-BLED and CHA_2_DS_2_-VASc scores were associated with NOACs use, whereas comorbidities including MI were associated with the use of warfarin.

In this study, we revealed that OACs are still being underused despite the introduction of NOACs. Treatment patterns of antithrombotics and the clinical factors associated with the choice of antithrombotics were similar before and after the introduction of NOACs. The clinical factors associated with the choice between taking warfarin and antiplatelets were similar to those associated with the choice between taking NOACs and antiplatelets.

In the present study, comorbidities affected the treatment pattern of antithrombotics both before and after the introduction of NOACs. AF patients with stroke were more likely to be prescribed OAC compared to antiplatelets, whereas AF patients with MI and PAD were more likely to use antiplatelets than OAC. The results of the present study are in line with recent real-world data analyses of the American College of Cardiology PINNACLE registry, which showed that in a cardiology outpatient population of NVAF patients with moderate to high risk of stroke, more than one-third were treated with aspirin alone, without OACs (12). In this study, the presence of CVD risk factors (e.g., hypertension and dyslipidemia) and CHD (e.g., prior MI/angina and recent coronary artery bypass graft) was associated with aspirin monotherapy, whereas prior stroke, TIA, and SE were associated with more frequent prescriptions of OACs. Therefore, based on these findings we can infer that treatment patterns are influenced by comorbidities of individual patients. The neurologists who take care of stroke patients are more likely to prescribe OACs because AF is associated with strokes with an increased risk of severe disability and mortality, and appropriate use of OAC is the most important modifiable factor of prognoses after stroke in patients with AF (13). However, treatment at a non-neurological department has been shown to be one of the factors associated with reluctance in prescribing OACs in patients with AF who have suffered a stroke (14). Ischemic events targeted by physicians may differ depending on patients' comorbidities. In several randomized clinical trials of ticagrelor, an antiplatelet agent that blocks the ADP (P2Y_12_) receptor, the most common type of recurrent ischemic event was reported to be stroke in patients with previous acute stroke/TIA, whereas CHD and limb revascularization were the most common in those with prior MI and PAD, respectively (15–17).

It is well-known that OACs—both warfarin and NOACs—are less likely to be used in women with AF (18, 19). A recent cohort study enrolling 2.3 million U.S. patients with a new diagnosis of AF and CHA_2_DS_2_-VASc score ≥2 showed that women, compared to men, were less likely to receive OAC which mediated the increased risk of stroke and decreased risk of intracranial hemorrhage (20). Low preference of OAC use in women by both physicians and patients (20) may explain why women were more likely to be prescribed antiplatelet agents than OACs in our study. In sensitivity analysis, we stratified logistic regression models by gender to explore the difference in factors affecting the choice of antithrombotics between male and female patients. The results of sensitivity analysis were generally comparable to that of main analysis, suggesting the factors affecting the choice of antithrombotics are generally similar between male and female patients (Supplementary Figure 3). Statin, which was another significant factor associated with OAC use, is the cornerstone of secondary prevention for vascular events in patients with coronary artery disease and PAD. Therefore, it is likely that statin treatment as well as antithrombotic prescription were influenced by the patients' comorbidities.

The choice between taking NOACs instead of warfarin was influenced by the CHA_2_DS_2_-VASc and HAS-BLED scores, albeit at small effect sizes. This finding was expected because unlike warfarin, NOACs do not require anticoagulation monitoring and demonstrated a clear reduction of risk of stroke and bleeding (21, 22). When compared with NOAC, warfarin use was associated with underlying renal disease in our study. With limited evidence of NOAC use in AF patients with chronic kidney disease, it is understandable that NOAC was less preferred in patients with renal disease. Several other factors were shown to have statistically significant association with either warfarin (MI, bleeding, hypertension, diabetes, congestive heart failure, COPD and digoxin use) or NOAC (age, CCI score, NSAID, PPI, H2-receptor antagonist, and antiarrhythmics use) prescription when compared to each other in our study, however these differences may not necessarily relate to clinical significance.

Further studies are needed to evaluate the role of combining NOACs with antiplatelets in patients with NVAF and the effect of comorbidities. Although the concomitant use of antiplatelets and OACs increases the risk of MB, a meta-analysis of randomized trials showed that it may be safer and more effective in preventing S/SE to use NOACs with concomitant aspirin therapy over warfarin in patients with NVAF (23). Randomized trials on the use of NOACs with antiplatelets have been conducted in patients with AF who underwent PCI (PIONEER AF PCI for rivaroxaban (24), RE-DUAL-PCI for dabigatran (25), AUGUSTUS for apixaban (26), and ENTRUST-AF PCI for edoxaban (27). In the AUGUSTUS trial, patients with NVAF and acute coronary syndrome or PCI treated with apixaban and a P2Y_12_ inhibitor showed lesser bleeding and fewer hospitalizations than those treated with warfarin and dual antiplatelets (26). Furthermore, a recent meta-analysis showed that NOACs were associated with less MB and fewer major cardiovascular adverse events, although warfarin was associated with lower rates of mortality and stroke (28). With additional trials of combined antiplatelets and NOACs in patients with comorbidities, treatment patterns can be changed accordingly.

Our study has some limitations. First, we did not consider variables that were not included in the HIRA database but may be associated with treatment patterns, such as clinical laboratory data, over-the-counter medications, and antithrombotic treatment preferences of patients and physicians. In addition, we assumed that patients complied with their treatment as prescribed. Second, as AF was defined based on the diagnosis codes, there is a possibility that misclassifications occurred during the identification of the study population. However, the proportion of misclassified patients is likely to be negligible given that we also included antithrombotic prescription and CHA_2_DS_2_-VASc score as inclusion criteria. Third, comparing two patient groups identified during two intake periods (i.e., before and after the introduction of NOACs) may not have been appropriate because there could be duplicate patients in both groups. However, we allowed duplication in patients because the aim of this study was to explore the prevalence, not the incidence, of antithrombotic treatment. This also provides an information on whether the treatment pattern changes within the same patient group after the introduction of NOACs. Given that the results were robust when we removed the duplicate patients in both intake periods, the impact of allowing duplicate patients might be negligible in this study. Lastly, it may not be the optimal time to observe the change in treatment pattern of antithrombotics immediately after the introduction of NOACs. Further studies are needed on treatment pattern of antithrombotics with a more recent data.

## Conclusion

In a large, real-world population of NVAF patients with moderate to high risk of S/SE, more than half were not treated with OACs, regardless of the introduction of NOACs. The treatment pattern of antithrombotics did not change following the introduction of NOACs. However, comorbidities had a considerable influence on the treatment pattern during both the “warfarin era” and “NOAC era.” Further clinical trials of NOACs in patients with comorbidities are needed.

## Data Availability Statement

The data analyzed in this study was obtained from the Korea Health Insurance Review & Assessment Service (HIRA) claims database, the following licenses/restrictions apply: requests to access these datasets must first be approved by the HIRA Service. Requests to access these datasets should be directed to the HIRA Service, opendata.hira.or.kr.

## Author Contributions

OYB, SKi, and HSS: conceptualization and writing—original draft preparation. OYB, SKi, YKO, M-YL, S-WJ, SH, HSS, and Y-HK: investigation. OYB, SKi, YKO, M-YL, S-WJ, SH, JR, SKa, HSS, and Y-HK: writing—review and editing. SKi and HSS: data curation, methodology, and formal analysis. JR and SKa: project administration. SKa, HSS, and Y-HK: supervision. All authors contributed to the article and approved the submitted version.

## Funding

This study was sponsored by Pfizer and Bristol Myers Squibb.

## Conflict of Interest

JR and SKa were employed by Pfizer Korea Ltd. This study received funding from Pfizer and Bristol Myers Squibb. The funders had the following involvement with the study: design, analysis, data interpretation, writing manuscript, and decision to submit for publication. OYB, YKO, M-YL, S-WJ, SH, SKi, HSS, and Y-HK were paid consultants to Pfizer and Bristol Myers Squibb in connection with the development of this manuscript.

## Publisher's Note

All claims expressed in this article are solely those of the authors and do not necessarily represent those of their affiliated organizations, or those of the publisher, the editors and the reviewers. Any product that may be evaluated in this article, or claim that may be made by its manufacturer, is not guaranteed or endorsed by the publisher.

## Abbreviations

MI: myocardial infarction
NOAC: non-vitamin K antagonist oral anticoagulant
NVAF: non-valvular atrial fibrillation
OAC: oral anticoagulant
OR: odds ratio
PAD: peripheral artery disease
PCI: percutaneous coronary intervention.
