# Supplementary material for: Comorbidities and Antithrombotic Treatment Pattern in Patients With Atrial Fibrillation
Source: Front Neurol. 2022 Mar 4;13:761603. doi: 10.3389/fneur.2022.761603 (PMC8931391; doi:10.3389/fneur.2022.761603)
Supplement: Supplementary file 1 [file Data_Sheet_1.PDF]

Supplemental Table I. Diagnosis, procedure, and medication codes used to define the study population

Category	Type of code	Code
Inclusion criteria		
Antithrombotics	Medication code	• Apixaban: 617001ATB, 617002ATB<br>• Dabigatran: 613701ACH, 613702ACH, 613703ACH<br>• Edoxaban: 643601ATB, 643602ATB, 643603ATB<br>• Rivaroxaban: 511401ATB, 511402ATB, 511403ATB, 511404ATB<br>• Warfarin: 249101ATB, 249102ATB, 249103ATB, 249104ATB, 249105ATB, 249106ATB, 249107ATB, 249108ATB, 249109ATB<br>• Aspirin: 110701ATE, 110705ACE, 111001ACE, 111001ATB, 111001ATE, 111002ATE, 111003ACE, 111003ATE<br>• Clopidogrel: 136901ATB, 492501ATB, 495201ATB, 498801ATB, 501501ATB<br>• Aspirin/clopidogrel: 517900ACE, 517900ACH, 517900ATE<br>• Prasugrel: 597301ATB, 597302ATB<br>• Ticagrelor: 615901ATB, 615902ATB<br>• Ticlopidine: 239201ATB, 239202ATB
AF	ICD-10 code[1)]	I48

CHA_2DS_2-VASc score	ICD-10 code[1)]	• Congestive heart failure: I50<br>• Hypertension: I10-I15<br>• Diabetes mellitus: E10-E14<br>• Stroke: I63, I693, G459<br>• Vascular disease: I21, I252, I70-I73
Exclusion criteria		
Valvular AF/ prosthetic heart valves	ICD-10 code[1)]	I05, I08, I09, I34, Q23, T820, T826, Z952, Z953, Z954
	Procedure code	M6580, M6581, M6582, O1791, O1792, O1793, O1794, O1795, O1796, O1797, O1798, O1799, M6531, M6532, M6533, O1690, O1730, 1740, O1750, O1760, O1770, O1781, O1782, O1783, O1810, O1826
Venous thromboembolism	ICD-10 code[1)]	I636, I676, I801, I802, I803, I808, I809, I81, I822, I823, I829, I26
Hip or knee replacement	Procedure code	N0711, N0715, N1711, N1715, N1721, N1725, N2070, N2072, N2077, N2710, N2712, N2717, N3710, N3712, N3717, N3720, N3722, N3727, N4710, N4712, N4717, N4720, N4722, N4727
End-stage of chronic kidney disease/ dialysis/ kidney transplant / pericarditis	ICD-10 code[1)]	N185, T824, Y602, Y612, Y622, Y841, Z49, Z992, E1022, E1122, E1222, E1322, E1422, Z940, I30, I31, I32
Transient AF or cardiac surgery	ICD-10 code[1)]	E05, I422

Procedure code	M5880, M6540, M6542, M6545, M6547, M6511

Abbreviations: AF, atrial fibrillation; ICD-10, International Classification of Diseases, Tenth Revision.

[1)] **Main and all subdiagnosis codes were used.**

Supplemental Table II. HAS-BLED score

Category	ICD-10 code[1)]	Point
Hypertension	I10-I15	1
Abnormal renal function	N183, N184	1
Abnormal liver function	B15, B16, B17, B18, B19, C22, K70, K71, K72, K73, K74, K75, K76, K77, D684, I982, I983, Z944	1
Stroke	I63, I693, G459	1
Bleeding history or predisposition	• Intracranial hemorrhage: I60, I61, I62, I690, I691, I692, S064, S065, S066, S068<br>AND procedure code for brain CT/MRI (HA441, HA451, HA461, HA471, HA851, HE101, HE102, HE135, HE201, HE202, HE235, HE301, HE302, HE401, HE402, HE501, HE502, HE535)<br>AND hospitalization<br>• Gastrointestinal bleeding: I850, I983, K2211, K226, K228, K250, K252, K254, K256, K260, K262, K264, K266, K270, K272, K274, K276, K280, K282, K284, K286, K290, K3181, K5521, K625, K920, K921, K922<br>• Other bleeding: D62, H448, H3572, H356, H313, H210, H113, H052, H470, H431, I312, N020-N029, N421, N831, N857, N920, N923, N930, N938, N939, M250, R233, R040, R041, R042, R048, R049, T792, T810, N950, R310, R311, R318, R58, T455, Y442, D683<br>• Transfusion (procedure code): X1001, X1002, X2011, X2012, X2021, X2022, X2031, X2032, X2041,	1

	X2042, X2051, X2052, X2061, X2062, X2071, X2072, X2081, X2082, X2091, X2092, X2101, X2102, X2111, X2112, X2121, X2122, X2131, X2132, X2141, X2142, X3010	
Age > 65	-	1
Antiplatelet or NSAID use	• Antiplatelets: 110701ATE, 110705ACE, 111001ACE, 111001ATB, 111001ATE, 111002ATE, 111003ACE, 111003ATE, 110706ATB, 110702ATB, 110902BIJ, 110802ATB, 110701ATB, 254300ACH, 394401ATB, 110704ATB, 110801ATB, 136901ATB, 492501ATB, 495201ATB, 498801ATB, 501501ATB, 517900ACE, 517900ACH, 517900ATE, 100431BIJ, 100430BIJ, 133201ACR, 133201ATB, 133201ATD, 133202APD, 133202ATB, 133202ATD, 133203ACR, 133203ATR, 147201ATB, 147202BIJ, 147203ATB, 597301ATB, 597302ATB, 615901ATB, 615902ATB, 239201ATB, 239202ATB, 240230BIJ	1
Alcoholism	F10, K70, X45, X65, Y15, Y90, Y91, E244, G312, G621, G721, I426, K292, K860, O354, P043, Q860, T510, Z502, Z714, Z721	1

Abbreviations: ICD-10, International Classification of Diseases, Tenth Revision; NSAID, nonsteroidal anti-inflammatory drugs.

[1)] **Main and all subdiagnosis codes were used.**

Supplemental Table III. Charlson Comorbidity Index

Category	ICD-10 code[1)]	Point
Cerebrovascular disease	G45, G46, H340, I60-I69	1
Congestive heart failure	I099, I110, I130, I132, I255, I420, I425-I429, I43, I50, P290	1
Chronic pulmonary disease	I278, I279, J40–J47, J60–J67, J684, J701, J703	1
Dementia	F00–F03, F051, G30, G311	1
Diabetes without chronic complication	E100, E101, E106, E108, E109, E110, E111, E116, E118, E119, E120, E121, E126, E128, E129, E130, E131, E136, E138, E139, E140, E141, E146, E148, E149	1
Mild liver disease	B18, K700-K703, K709, K713-K715, K717, K73, K74, K760, K762-K764, K768, K769, Z944	1
Myocardial infarction	I21, I22, I252	1
Peripheral vascular disease	I70, I71, I731, I738, I739, I771, I790, I792, K551, K558, K559, Z958, Z959	1
Peptic ulcer disease	K25–K28	1
Rheumatologic disease	M05, M06, M32–M34 M315M351, M353, M360	1
Diabetes with chronic complication	E102–E105, E107, E112–E115, E117, E122-E125, E127, E132–E135, E137, E142–E145, E147	2

Hemiplegia or paraplegia	G041, G114, G801, G802, G81, G82, G830-G834, G839	2
Any malignancy, including leukemia and lymphoma	C00–C26, C30–C34, C37–C41, C43, C45–C58, C60–C76, C81–C85, C88, C90–C97,	2
Renal disease	I120, I131, N032-N037, N052-N057, N18, N19, N250, Z490-Z492, Z940, Z992	2
Moderate or severe liver disease	I850, I859, I864, I982, K704, K711, K721, K729, K765, K766, K767	3
AIDS/HIV	B20–B22, B24	6
Metastatic solid tumor	C77–C80	6

Abbreviations: AIDS, acquired immunodeficiency syndrome; HIV, human immunodeficiency virus; ICD-10, International Classification of Diseases, Tenth Revision.

[1)] Main and all subdiagnosis codes were used.

Supplemental Figure I. Treatment patterns of antithrombotics before and after the introduction of NOACs without duplicate patients who were included in both intake periods

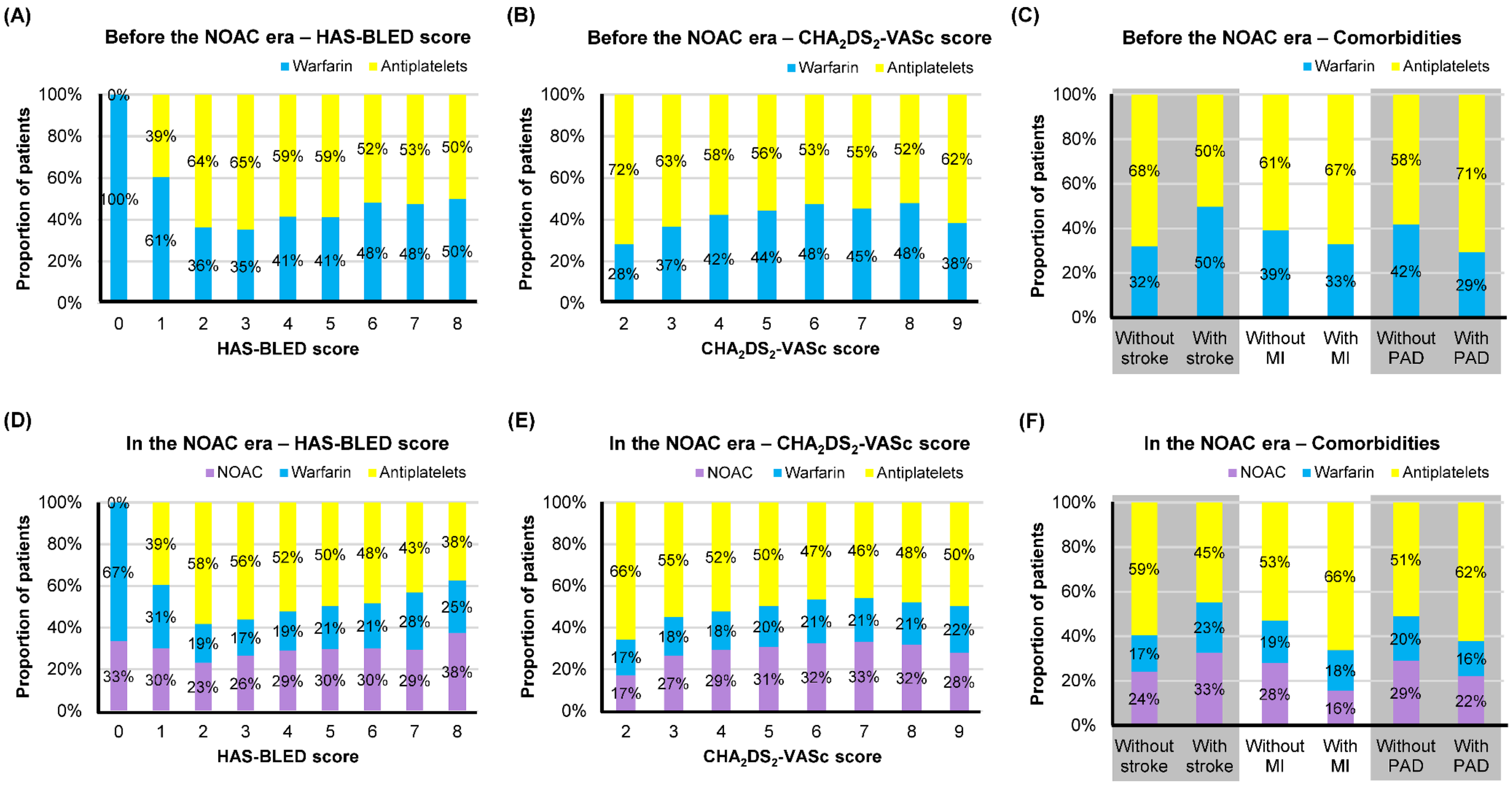

Proportions of patients prescribed each type of antithrombotics before the NOAC introduction **(A)** according to the HAS-BLED score; **(B)** according to the CHA_2DS_2-VASc score; **(C)** according to the comorbidities, and after the NOAC introduction **(D)** according to the HAS-BLED score; **(E)** according to the CHA_2DS_2-VASc score; **(F)** according to the comorbidities.

Abbreviations: MI, myocardial infarction; NOAC, non-vitamin K antagonist oral anticoagulants; PAD, peripheral artery disease.

Supplemental Figure II. Clinical factors associated with the choice of combined oral anticoagulant and antiplatelet therapy versus oral anticoagulant monotherapy

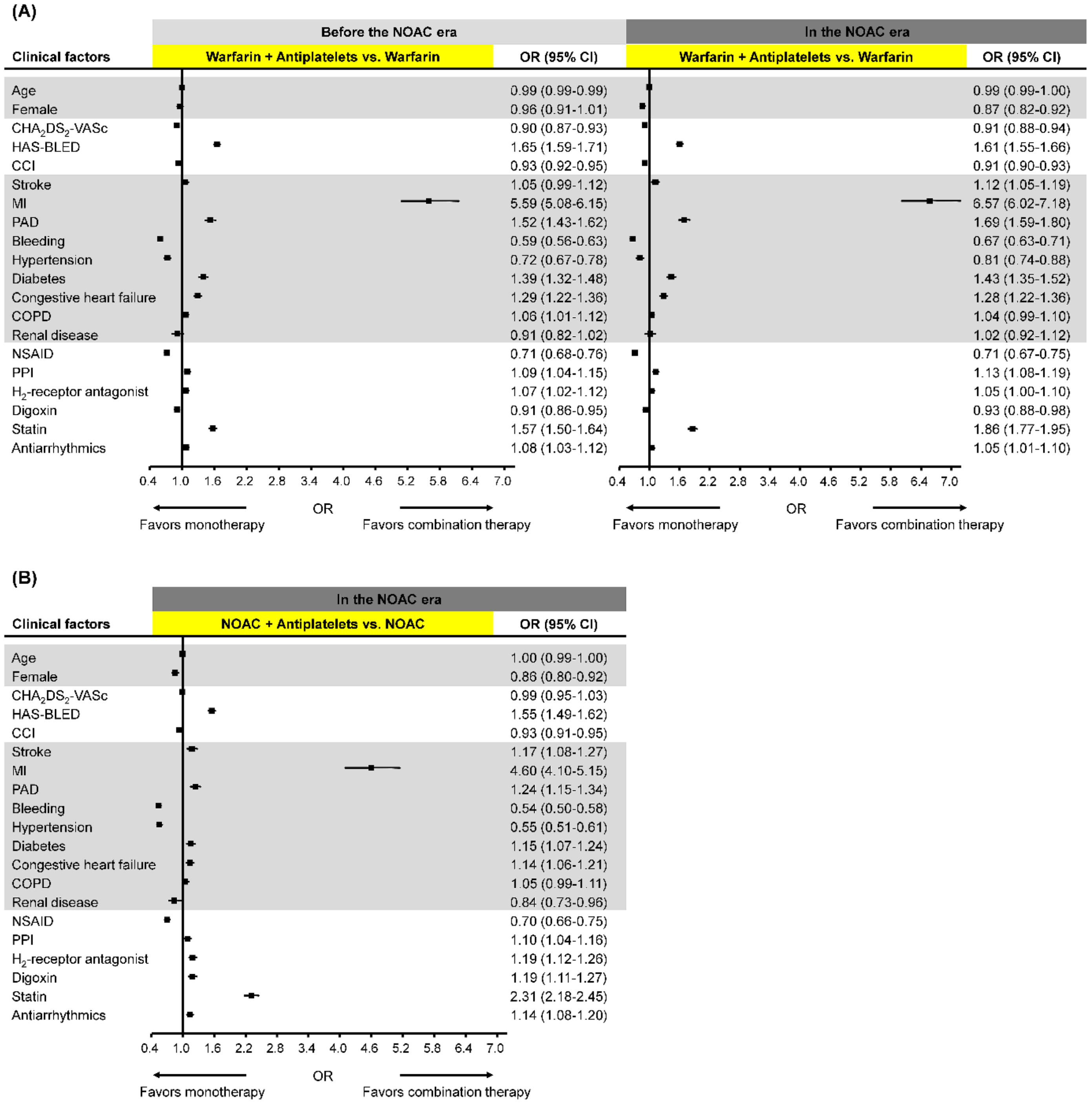

Odds ratios for the association of clinical factors with the choice of **(A)** warfarin + antiplatelets versus warfarin before and in the NOAC era; **(B)** NOAC + antiplatelets vs NOAC in the NOAC era.

Abbreviations: CCI, Charlson Comorbidity Index; CI, confidence interval; COPD, chronic obstructive pulmonary disease; MI, myocardial infarction; NOAC, non-vitamin K antagonist oral anticoagulants; NSAID, nonsteroidal anti-inflammatory drug; OR, odds ratio; PAD, peripheral artery disease; PPI, proton pump inhibitor.

Supplemental Figure III. Clinical factors associated with the choice of antithrombotics stratified by gender

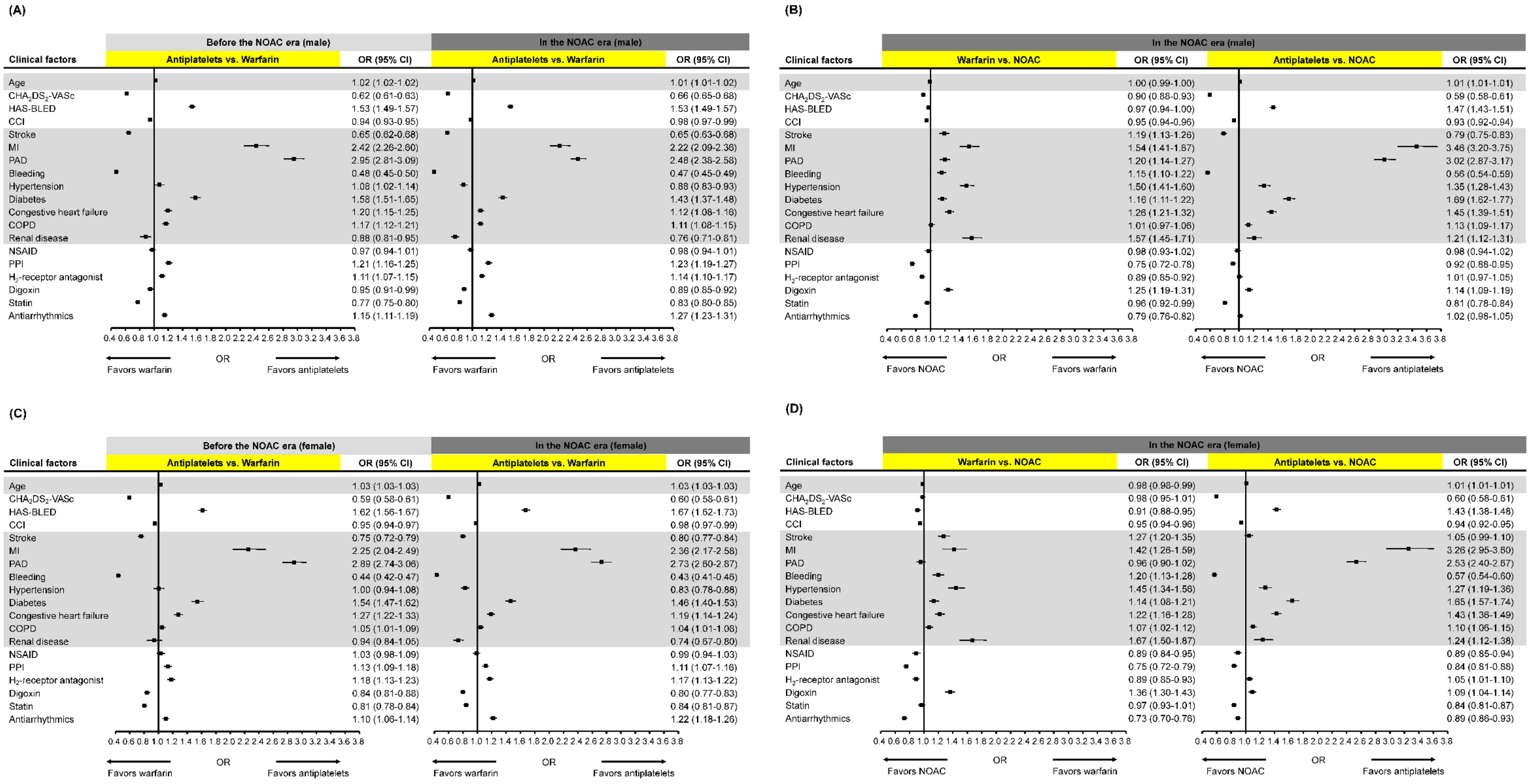

Odds ratios for the association of clinical factors with the choice of **(A)** antiplatelets versus warfarin before and in the NOAC era among male patients; **(B)** warfarin/antiplatelets versus NOAC in the NOAC era among male patients; **(C)** antiplatelets versus warfarin before and in the NOAC era among female patients; **(D)** warfarin/antiplatelets versus NOAC in the NOAC era among female patients.

Abbreviations: CCI, Charlson Comorbidity Index; CI, confidence interval; COPD, chronic obstructive pulmonary disease; MI, myocardial infarction; NOAC, non-vitamin K antagonist oral anticoagulants; NSAID, nonsteroidal anti-inflammatory drug; OR, odds ratio; PAD, peripheral artery disease; PPI, proton pump inhibitor.
